# Influence of Zr on Al-Ti-B-Based Grain Refiners in AlSiMgCuZr Alloy

**DOI:** 10.3390/ma18133000

**Published:** 2025-06-24

**Authors:** Dawid Kapinos, Bogusław Augustyn, Sonia Boczkal, Kamila Limanówka, Bartłomiej Płonka, Aldona Garbacz-Klempka, Marcin Piękoś, Janusz Kozana

**Affiliations:** 1 Łukasiewicz Research Network—Institute of Non-Ferrous Metals, Light Metals Centre, Piłsudskiego 19, 32-050 Skawina, Poland; dawid.kapinos@imn.lukasiewicz.gov.pl (D.K.); boguslaw.augustyn@imn.lukasiewicz.gov.pl (B.A.); sonia.boczkal@imn.lukasiewicz.gov.pl (S.B.); kamila.limanowka@imn.lukasiewicz.gov.pl (K.L.); bartlomiej.plonka@imn.lukasiewicz.gov.pl (B.P.); 2 Faculty of Foundry Engineering, AGH University of Krakow, Reymonta 23, 30-059 Cracow, Poland; mpiekos@agh.edu.pl (M.P.); jkozana@agh.edu.pl (J.K.)

**Keywords:** AlSiMgCuZr alloys, Zr poisoning, aluminium alloys, grain refinement

## Abstract

One of the most effective methods of improving the properties of aluminium alloys is grain refining using Al-Ti-B master alloys. In contrast, zirconium is a key alloying element, used mainly in 2xxx and 7xxx series aluminium alloys, where it contributes to dispersion enhancement and reduces the rate of dynamic recrystallisation. However, even trace amounts of zirconium—just a few hundredths of ppm—significantly reduce the performance of Al-Ti-B grain refiners, a phenomenon known as ‘Zr poisoning’. This study investigates the impact of holding time and the level of Al-5Ti-1B addition on the microstructure and properties of an AlMgSi(Cu) alloy containing 0.15 wt.% Zr, cast as 7-inch DC billets. The structure and phase distribution were characterised using optical microscopy (OM), scanning electron microscopy (SEM) with energy-dispersive spectroscopy (EDS), and transmission electron microscopy (TEM). Grain size and morphology were evaluated through macrostructure analysis (etched cross-sections and polarised light microscopy), while chemical and elemental distributions were analysed via SEM-EDS and STEM-EDS mapping. Additionally, Brinell hardness measurements were conducted across the billet diameter to assess the correlation between grain size and mechanical properties. The results show that reducing holding time and increasing the Al-5Ti-1B addition improves grain refinement efficiency despite the presence of Zr. The finest grain structure (150–170 μm) and most homogeneous hardness distribution were achieved when the grain refiner was continuously fed during casting at 80 ppm B. These findings are supported by the literature and contribute to a deeper understanding of the Zr poisoning effect and its mitigation through optimized casting practice.

## 1. Introduction

The addition of borides or titanium carbides has been effective in reducing grain size in aluminium alloy castings since the late 1940s [1,2]. A fine and homogeneous grain structure improves mechanical properties and reduces defects. By the 1950s, grain refiners based on Al-Ti-B compounds were widely used in industry. Al-5Ti-1B is the most commonly used grain refiner, containing soluble Al_3_Ti particles and insoluble TiB_2_ particles dispersed in an aluminium matrix. TiB_2_ particles act as highly active nucleation sites for heterogeneous α-Al solid-phase formation, promoting the development of fine-grain [3]. To date, most publications on grain refinement have not addressed the phenomenon of ‘Zr poisoning’. The first reports on the challenges of α-Al primary grain fragmentation using Al-Ti-B refiners in zirconium-containing alloys appeared as early as the 1970s and 1980s [4,5,6,7]. Despite decades of research on Zr poisoning, few studies have addressed the interplay of refiner feed strategy and contact time in DC casting of Zr-containing industrial Al alloys—an area this study aims to explore.

Jones and Pearson [4] demonstrated that achieving a grain size below 250 µm in Al-5Zn-1.5Mg-0.2Zr alloy required an increase in the level of grain refiner addition. They also noted that extended holding times led to an increase in grain size. Birch and co-authors [5,6] reported grain refiner poisoning in Al-5Ti-1B when used in commercial 7xxx alloys containing Zr. Similarly, Abdel-Hamid [7], Johnsson [8], and Kearns [9] observed Zr poisoning in alloys containing 0.1–0.15% Zr. Read [10] analysed grain refining rods and their effect on the structure of the AlZn5Mg1.5Zr0.2 alloy, demonstrating that an initial addition of 200 ppm Ti by AlTi5B1 reduced the grain size, but after 30 min, a grain coarsening occurred. The results suggest that during this period Zr diffusion into TiB_2_ took place. To mitigate this effect, it was recommended that AlTi5B bars be used only in the final stage before casting. Bunn et al. [11] found that 0.05% Zr reduces the performance of AlTi5B1, especially at elevated temperatures (760 °C and 800 °C). Spittle and Sadli [12] investigated the effect of Fe, Cr, Si, and Zr on the grain fineness of high purity aluminium (99.99%) compared to commercial purity aluminium (99.7–99.85%). They observed that Zr poisoning occurred only in the presence of Fe and Si after a 30 min holding period. This finding is supported by studies such as [13], which shows that Zr alone does not adversely affect the grain refining performance of Al-Ti-B, even if some Ti atoms are replaced by Zr atoms on the surface of TiB_2_ particles. In contrast, the co-precipitation effect occurs when a certain amount of Fe is present in commercial-grade aluminium alloys. The exact mechanism of the co-precipitation effect in Al-Ti-B grain refining remains unclear despite decades of research. It is generally accepted that the phenomenon may occur in the presence of alloying elements such as Zr, Cr, Si, and Li and is mainly due to the deactivation of nucleating particles, such as Al_3_Ti and TiB_2_, present in the Al-Ti-B master alloy [4,7,14,15,16,17,18]. Zr poisoning results from the chemical and structural modification of TiB_2_ particle surfaces, which impairs their ability to heterogeneously nucleate α-Al grains. The Zr poisoning mechanism can therefore be summarized as the dissolution of the two-dimensional Al_3_Ti (2DC) compound formed on the TiB_2_ surface and the formation of a Ti_2_Zr (2DC) atomic monolayer on the TiB_2_ substrate surface, replacing the original TiB_2_ surface. As a result, TiB_2_ particles become incapable of heterogeneous nucleation of α-Al due to increased atomic lattice mismatch [19]. This effect has also been confirmed by, Pang and others [20] who showed that that the addition of Zr creates bonds of a more ionic nature, and its diffusion into the interior of the particles is more difficult and slower; however, after penetrating the Al_3_Ti structure, Zr replaces Ti atoms in the second layer, which results in the formation of the Al_3_(Ti,Zr) phase, less active in nucleation. Due to the chemical modification of the surface and interior of Al_3_Ti particles and by reducing their energy activity, they contribute to the poisoning effect, i.e., a decrease in the efficiency of grain refining in aluminium alloys. Studies [4,11,15] have shown that the intensity of the poisoning effect increases over time, which is associated with the gradual replacement of titanium (Ti) atoms by zirconium (Zr) atoms, thus reducing the nucleation capacity of α-Al grains. This phenomenon is irreversible, unlike sedimentation caused by gravitational deposition of nucleating particles, which can be restored by stirring the melt.

To eliminate or mitigate this detrimental effect, intensive efforts have been undertaken. One approach is the potential use of Al-Ti-C-based grain refiners as an alternative solution to the Zr poisoning problem [21,22,23]. These studies suggest that Al-Ti-C exhibits some resistance to the negative effects of Zr in 7xxx series alloys. Other publications [24,25], however, have shown that Al-Ti-C may also undergo poisoning in the presence of Zr, indicating that Zr interacts with both TiAl_3_ and TiC phases, thereby limiting their effectiveness. The efficiency of Al-5Ti-0.4C grain refinement decreases when the Zr content exceeds 0.03 wt.%.

In recent years, to overcome the problem of aluminium grain refinement being “poisoned” by Si and Zr, Daoxiu Li and colleagues [26] developed a new Al–Ti–C–B grain refiner doped with a Ti–Si–Zr complex. This complex modifies the surface energy of TiB_2_ particles, enhancing their nucleation efficiency while suppressing the formation of detrimental Al_3_Zr/Al_3_Ti phases. As a result, the alloy exhibits a significantly finer microstructure and improved mechanical properties.

Fang and colleagues [27] demonstrated the synergistic effect of Zr (0.25%) and Y (0.3%) in an Al–Si (A356) alloy. The addition of Y modifies the eutectic silicon, while Zr effectively refines the α-Al grains through the formation of Al_3_(Y, Zr) particles.

The aim of the research presented in this paper was to evaluate the grain refining of AlMgSi(Cu) alloy with above standard copper content containing 0.15% Zr by optimising the addition of the modifier Al-5Ti-1B. The effect of the amount of grain refiner added and its exposure time on the microstructure and hardness distribution in the billets was studied in order to minimise the effect of Zr poisoning. This is a research area that is still evolving and deserves increased attention from the scientific community, as it represents a real and unresolved technological challenge. This issue is still unresolved and will be more extensively investigated in the coming years, given its significance for microstructural control and mechanical properties optimization in Zr-containing aluminium alloys. The present study contributes to this effort by providing experimental insights into refining efficiency and strategies for mitigating Zr-related degradation in industrially relevant alloy systems.

## 2. Materials and Methods

The AlSiMgCuZr alloys were prepared in an 800 kg capacity gas crucible furnace using aluminium billets. Once the temperature reached 740–750 °C, Si, Cu, Mn, Zr, Cr, and Mg were added as pure metals or reference alloys (Altab Mn75, Altab Cr75, and AlZr15). The melt was purified using an argon purge (10 L/min) for 15 min at approximately 740 °C, and a sample was taken for chemical composition determination on an ARL 4460 optical emission spectrometer, Thermo Fisher Scientific, Waltham, MA, USA (Tables 2–4).

Three different variants of grain refiner application were investigated, in which Al–5Ti–1B was added in different ways—either before or during casting and in different amounts:

- Billet 1—grain refiner was added to the furnace before the casting process, in an amount calculated to achieve 60 ppm B (0.0060% B), corresponding to 300 ppm Ti. The billets were cast at a length of 2 m.
- Billet 2—grain refiner fed continuously through the feeder during casting into the trough, added at 60 ppm B. The billets were cast to a length of 5 m.
- Billet 3—grain refiner fed continuously through the feeder during casting into the trough, added at 80 ppm B. The billets were cast to a length of 5 m.

These variants were selected to investigate whether a continuous feed during casting compensates for the time-dependent depletion of active Ti and B due to Zr poisoning.

Direct-cooled (DC) casting was carried out at the Łukasiewicz-IMN Skawina facility using three 178 mm (7″) diameter hot-top moulds with continuous lubrication. The metal was filtered through a 30 ppi ceramic filter. A representative view of the cast billets and casting parameters is shown in Table 1.

Samples taken from the beginning (1–3a) and the end (1–3b) of the billets were cut from the cross-sections for macrostructure observations. The macrostructure of the cross sections or billet samples was observed using Trucker’s reagent, which consists of hydrochloric acid, hydrofluoric acid, nitric acid and water. The structure of the etched billets was scanned. Microscopic observations were performed on samples approximately 3 cm in diameter, taken from mid-radius positions of the billet samples. The samples were then cut and ground to parallel surfaces using 4000 grit sandpaper, Struers and then polished on 1 µm grit diamond suspension cloths. Microstructural observations of the prepared metallographic samples were carried out using a Zeiss Axio Observer 7 Mat (Carl Zeiss, Oberkochen, Germany) metallographic light microscope equipped with dedicated Zen Core software. In order to make the grains visible, the samples were etched with Barker’s reagent, a mixture of tetrafluoroboric acid and distilled water. Observations were carried out under bright field and polarised light. The grain size was measured using the intercept method by measuring about 100 grains for each sample. The microstructure of the prepared metallographic samples was also investigated using the Inspect F50 (FEI Company, Hillsboro, OR, USA) scanning electron microscope (SEM) equipped with an emission gun (FEG) and energy-dispersive X-ray spectroscopy (EDS) system. Elemental distribution maps (mapping) were obtained for micro areas of 512 µm × 400 µm. Samples for SEM-EDS analysis were taken from 1/2 radius of the macrostructure samples. Additionally, the sample for the TEM was prepared from an ingot by casting a cut templet of about 2 kg by remelting in a high frequency laboratory induction furnace under controlled conditions and pouring the liquid metal through the filter of a Prefil Footprinter^®^ device, ABB, Saint-Laurent, QC, Canada. A thin film sample was then cut directly from above the filter, and by grinding and electropolishing both sides on a TENUPOL device in Struers A2, Cleveland, OH, USA reagent, the sample was prepared as a thin film. Observations were carried out on a Tecnai G20 (FEI Company, Hillsboro, OR, USA) X-twin transmission electron microscope ((S)TEM) with FEI HAADF and EDS attachment. Samples from the end of the ingots were cut from cross sections to determine the mechanical properties.

Hardness measurements in the cross-section of the billet were made using the Brinell hardness test (2.5 mm ball, 613 N load) on a Duramin 2500 Brinell Hardness Tester (Struers, Copenhagen, Denmark). Measurements were taken at 10 mm intervals, with additional measurements at 1–2 mm intervals near the edge of the billet. A static tensile test of the billet was performed in accordance with the requirements of the standard PN-EN ISO 6892-1:2020-05 [28] on an Instron 5582, Norwood, MA, USA—max load 100 kN. Strain de-formation was measured with an extremely accurate video extensometer (Instron, Norwood, MA, USA), with crosshead speed 1 0.37 mm/min to determine yield strength and crosshead speed 2 3 mm/min to the end of test. The gauge length was 25 mm.

## 3. Results

Table 2, Table 3 and Table 4 summarize the chemical composition of the billets before and during casting.

**Table 2:** Chemical composition of the billet 1 (length: 2 m) [wt.%].

	Si	Fe	Cu	Mn	Mg	Cr	Zn	Ti	B	Zr
Before casting	1.03	0.11	0.63	0.60	0.71	0.26	0.018	0.031	0.005	0.15
1 m	1.00	0.11	0.61	0.59	0.69	0.27	0.022	0.027	0.003	0.15
2 m	1.00	0.11	0.61	0.60	0.71	0.26	0.021	0.022	0.002	0.15
Average	1.02	0.11	0.61	0.60	0.70	0.27	0.021	0.024	0.003	0.15

**Table 3:** Chemical composition of the billet 2 (length: 5 m) [wt.%].

	Si	Fe	Cu	Mn	Mg	Cr	Zn	Ti	B	Zr
Before casting	1.07	0.07	0.67	0.55	0.67	0.27	0.023	0.013	0.000	0.15
1 m	1.09	0.08	0.68	0.55	0.67	0.27	0.023	0.027	0.003	0.15
2 m	1.11	0.08	0.69	0.55	0.68	0.27	0.023	0.026	0.003	0.15
3 m	1.11	0.08	0.68	0.55	0.67	0.27	0.023	0.027	0.003	0.15
4 m	1.07	0.08	0.67	0.55	0.66	0.27	0.022	0.026	0.003	0.14
4.5 m	1.07	0.08	0.66	0.56	0.66	0.27	0.023	0.031	0.004	0.15
Average	1.09	0.08	0.68	0.55	0.67	0.27	0.023	0.027	0.003	0.15

**Table 4:** Chemical composition of the billet 3 (length: 5 m) [wt.%].

	Si	Fe	Cu	Mn	Mg	Cr	Zn	Ti	B	Zr
Before casting	1.02	0.08	0.60	0.63	0.69	0.26	0.024	0.020	0.002	0.16
1 m	1.03	0.09	0.62	0.61	0.70	0.26	0.025	0.035	0.005	0.15
2 m	1.03	0.09	0.59	0.62	0.68	0.26	0.024	0.035	0.005	0.16
3 m	1.02	0.08	0.60	0.62	0.68	0.25	0.022	0.037	0.005	0.15
4 m	1.01	0.09	0.59	0.62	0.67	0.25	0.022	0.036	0.005	0.15
4.5 m	1.04	0.09	0.61	0.62	0.69	0.25	0.021	0.041	0.006	0.15
Average	1.03	0.09	0.60	0.62	0.68	0.26	0.023	0.036	0.005	0.15

In variant 1, where a grain refiner was added before casting at a concentration of 60 ppm B (Table 2), the Ti and B content decreased with time. The time from the addition of the grain refiner to the casting of the 2 m billet was about 35 min. For variant 2, the melt initially contained 130 ppm Ti and 4 ppm B. During casting, grain refiner was added in an amount to increase the content to 460 ppm Ti and 60 ppm B. However, the Ti and B content during casting remained constant but lower (260–310 ppm Ti and 30 ppm B) compared to the expected values (Table 3). A similar trend was observed for billet 3, where the expected content was 600 ppm Ti and 80 ppm B, but the measured values were 350–410 ppm Ti and 50 ppm B (Table 4).

**Figure 1:**
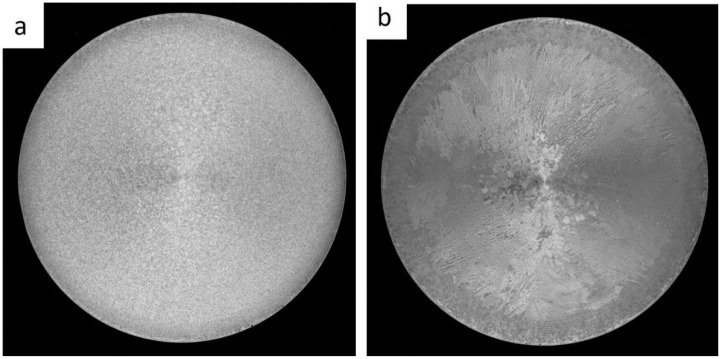
Macrostructure of billet 1 with grain refiner added before casting (60 ppm TiB_2_), with samples taken from the beginning (**a**) and the end (**b**) of the billet.

**Figure 2:**
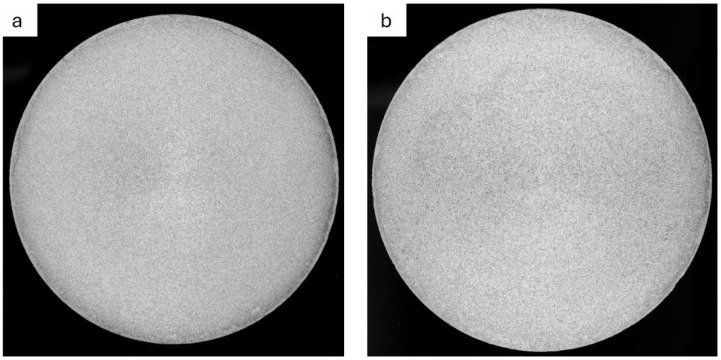
Macrostructure of billet 2 with grain refiner added during casting (60 ppm TiB_2_), with samples taken from the beginning (**a**) and the end (**b**) of the billet.

**Figure 3:**
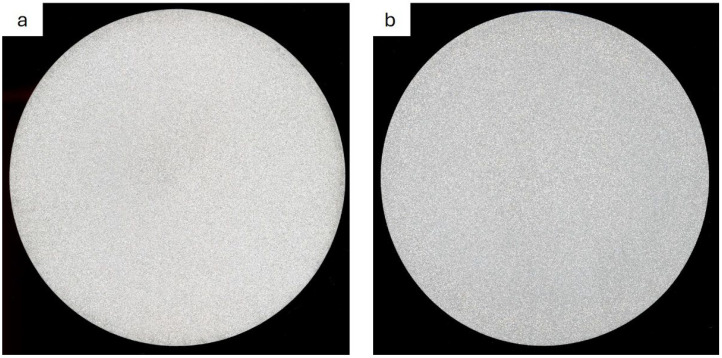
Macrostructure of billet 3 with grain refiner added during casting (80 ppm TiB_2_), with samples taken from the beginning (**a**) and the end (**b**) of the billet.

To investigate the effect of the grain refiner on grain size, macrostructure analysis was carried out on samples cut from the cross-section at the beginning and end of the billet. The sample taken from the beginning of billet 1 showed a fine-grained structure (Figure 1), while the sample from the end of billet 1 showed larger, more differentiated grains that were both elongated and equiangular in the central part of the billet. Billets 2 and 3 were observed to exhibit a fine-grained equiangular structure throughout (Figure 2 and Figure 3).

**Figure 4:**
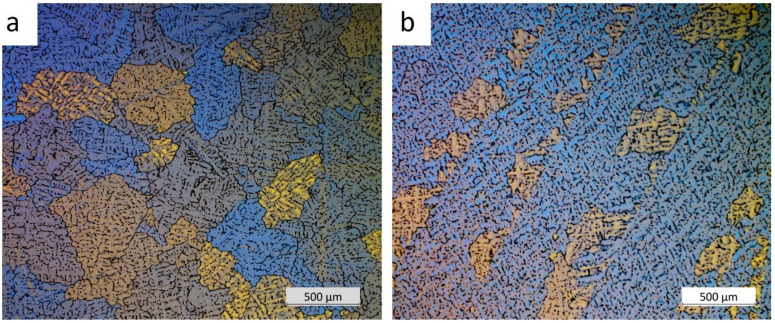
Grain structure in billet 1 with grain refiner added before casting (60 ppm TiB_2_), with samples taken from the beginning (**a**) and the end (**b**) of the billet.

**Figure 5:**
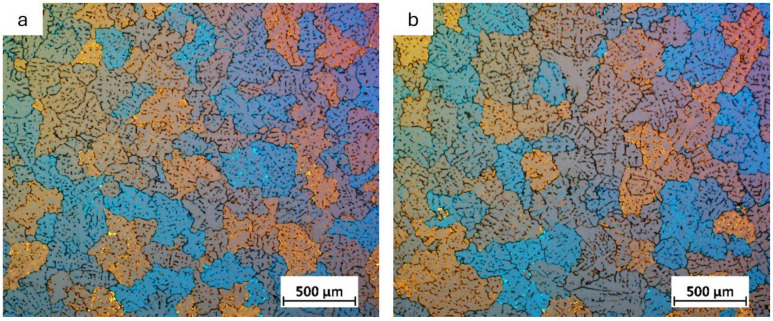
Grain structure in billet 2 with grain refiner added during casting (60 ppm TiB_2_), with samples taken from the beginning (**a**) and the end (**b**) of the billet.

**Figure 6:**
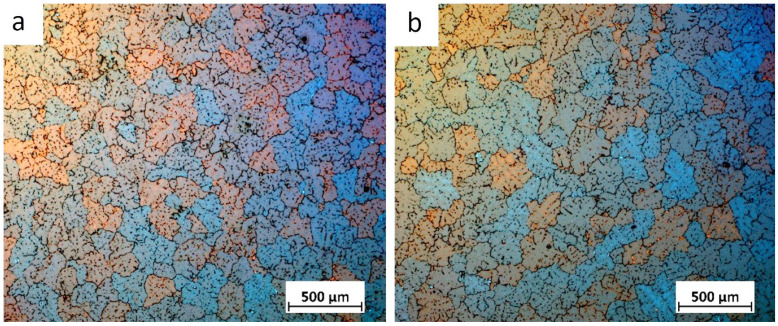
Grain structure in billet 3 with grain refiner added during casting (80 ppm TiB_2_), with samples taken from the beginning (**a**) and the end (**b**) of the billet.

**Figure 7:**
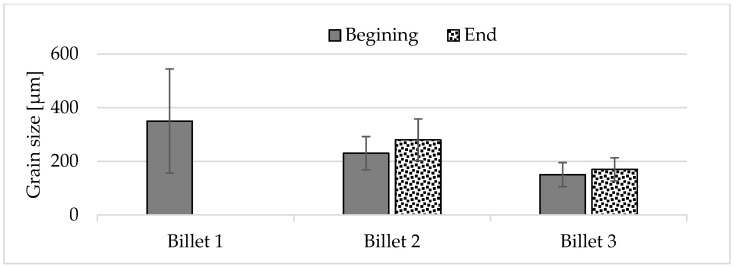
Average grain size at the beginning and end of the billet.

For a detailed analysis of the grain size, microstructural observations of the billets were carried out after etching with Barker’s reagent. The results of these observations are shown in Figure 4, Figure 5, Figure 6 and Figure 7.

The grain size at the beginning of sample 1 was approximately 350 μm, whereas the grains at the end of the sample were so large that their size could not be accurately determined using light microscopy. It was only possible to estimate that the average grain size ranged from 1 to 6 mm. Observations of the grain indicated that the billets 2 and 3 exhibited a predominantly equiaxial quasi-dendritic grain structure. The average grain size in billet 2 ranged from 230 to 280 μm, while in the billet 3 it ranged from 150 to 170 μm.

**Figure 8:**
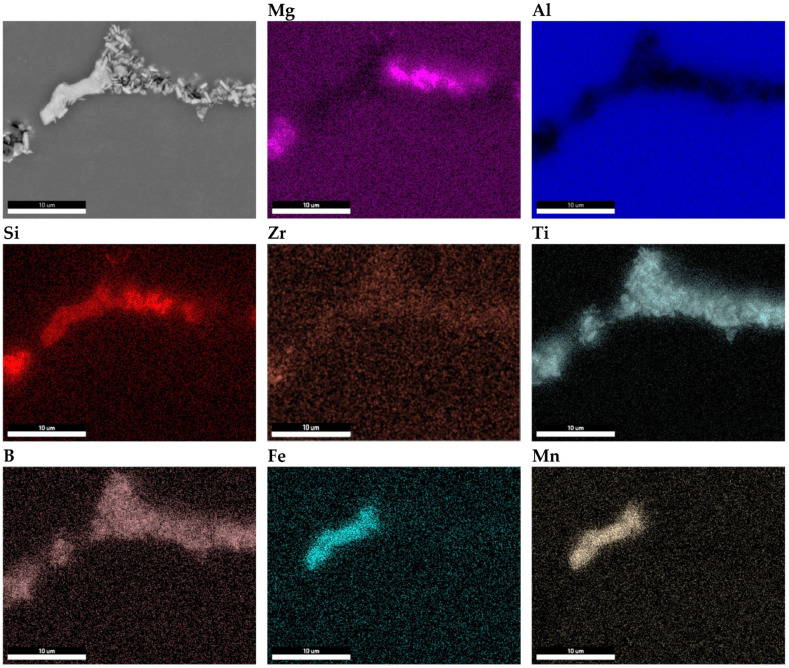
SEM-EDS map of the distribution of chemical elements in billet 1 with grain refiner added before casting (60 ppm TiB_2_).

The sample taken from the end of the billet 1 was further examined using a scanning electron microscope (SEM). The results of these observations are presented in Figure 8.

Clusters of metallic compounds other than the naturally occurring phases in the alloy were observed along the grain boundaries. The elemental distribution maps reveal local enrichments of Zr, Ti, and B in regions containing iron-rich phases (AlFeMnSi) and Mg_2_Si phases, which are naturally present in this type of alloy (Figure 8).

**Figure 9:**
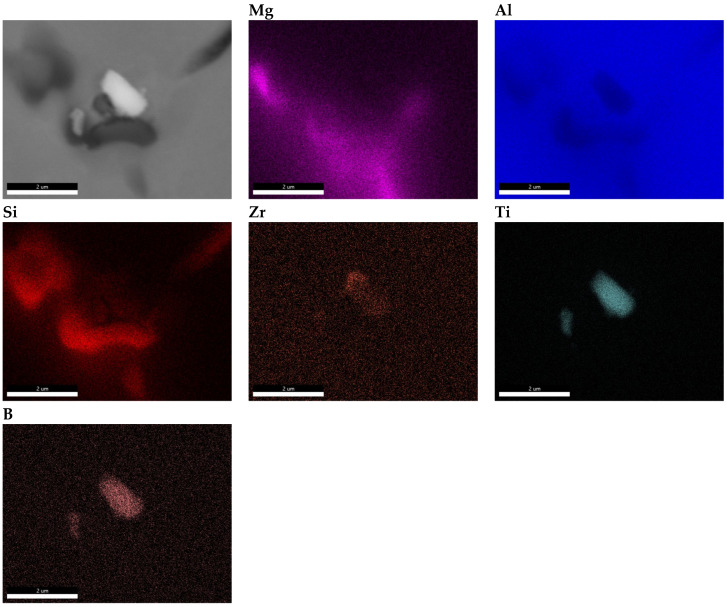
SEM-EDS map of the distribution of chemical elements in billet 3 with grain refiner added during casting (80 ppm TiB_2_).

The sample taken from the end of billet 3 was also examined using a scanning electron microscope (SEM). The results of these observations are shown in Figure 9.

No TiB_2_ clusters were observed in the examined billet, and the grains were evenly distributed throughout the billet volume. Isolated TiB_2_ particles in combination with Zr were detected, as shown in Figure 9. However, this did not affect the grinding efficiency, as no TiB_2_ clusters were observed, unlike in billet 1.

**Figure 10:**
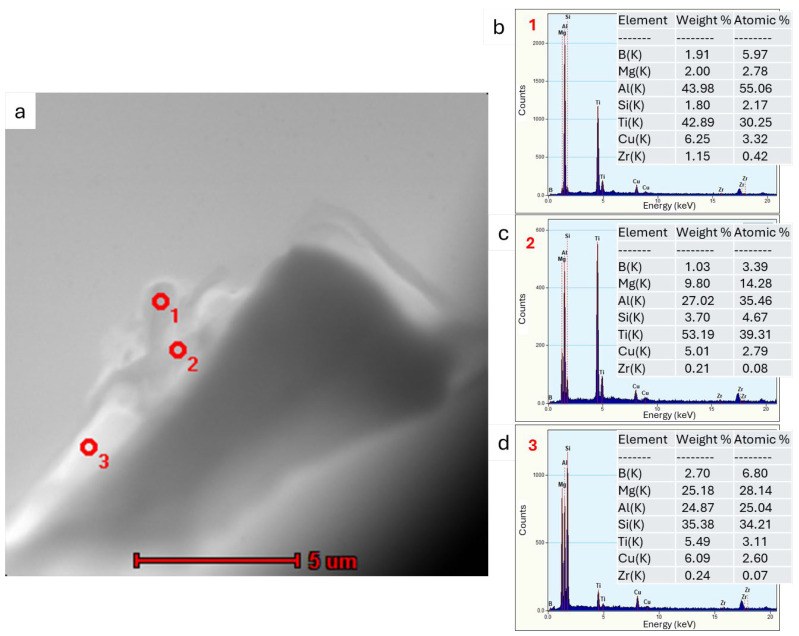
STEM analysis (**a**) together with spot EDS chemical analysis in the microareas shown in the graphs for (**b**) point 1, (**c**) 2, and (**d**) 3.

**Figure 11:**
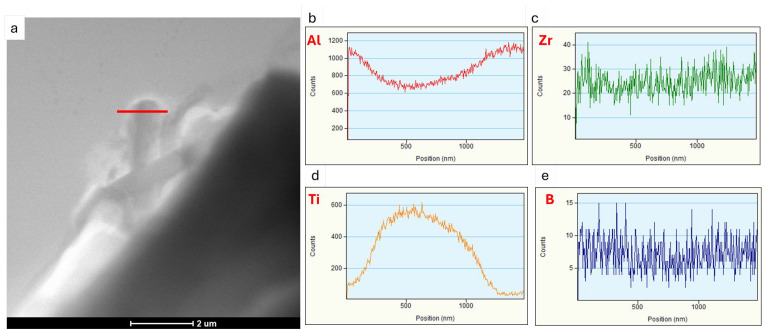
STEM analysis from the Mg_2_Si phase region (**a**) together with linear EDS chemical analysis by particle shown in the graphs for (**b**)Al, (**c**) Ti, (**d**) Zr and (**e**) B.

**Figure 12:**
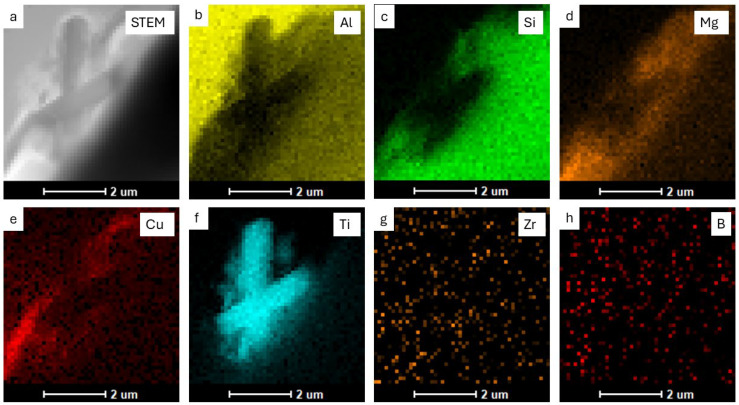
STEM image (**a**) of the particle area together with elemental distribution maps of (**b**) Al, (**c**) Si, (**d**) Mg, (**e**) Cu, (**f**) Ti, (**g**) Zr, (**h**) B.

The sample was also examined using a transmission electron microscope (TEM). The results of these observations are shown in Figure 10, Figure 11 and Figure 12.

Transmission electron microscopy analyses revealed a small proportion of phases containing TiB_2_ particles at the boundary. From the observation of the area where particles containing elements such as Ti, B and Zr were found, point and linear micro-area chemical analyses and elemental distribution maps were carried out (Figure 10, Figure 11 and Figure 12). The point chemical composition analyses show that the particle containing Ti and B (Figure 10) also has an elevated Zr content. The analysis in the surroundings of the Mg_2_Si phase, especially at point 3 (Figure 10c), revealed trace amounts of Ti and Zr. Linear analysis across the TiB_2_ particle confirmed the presence of Zr (Figure 11). Slight increases in Zr intensity (Figure 11d) were observed at the beginning and end of the particle, in line with the Ti intensity profile (Figure 11c). The B profile (Figure 11e) was characterised by similar small intensity peaks, but the B intensity decreased in the inner part of the particle. The elemental distribution maps (Figure 12) confirm the presence of TiB_2_ particles in the form of 2 to 3 μm rods deposited near the Mg_2_Si phase.

**Figure 13:**
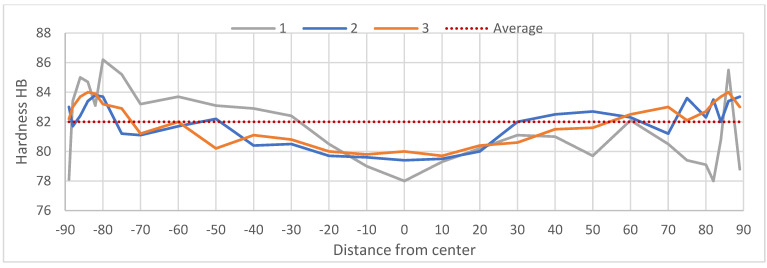
Brinell hardness distribution across the billet diameter.

**Figure 14:**
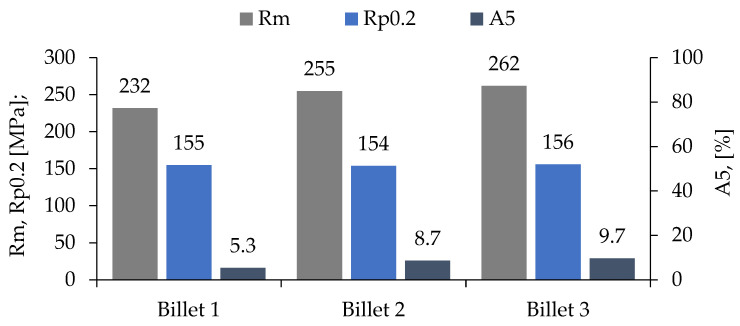
Tensile strength of cast billet.

To compare the influence of grain size on mechanical properties, the tensile strength and hardness distribution measurements were conducted along the diameter of the billet’s cross-section in the as-cast (F) condition. The hardness distribution across the diameter of cast billets is presented in Figure 13 while Figure 14 present the results of the mechanical properties tests of the ends of cast billets having different divisions.

Brinell hardness measurements (Figure 13) revealed that billets 2 and 3 exhibited a stable hardness distribution (±2 HB), whereas billet 1 showed greater fluctuations (±4 HB), which can be attributed to its inhomogeneous structure.

Billet 1, with a coarse-grained microstructure (Figure 1b and Figure 4b), exhibited the lowest values of tensile strength (232 MPa) and elongation (5.3%), while maintaining a comparable yield strength to the other samples (Figure 14). Billet 2, with finer grains (Figure 2b and Figure 5b, i.e., 280 μm), showed an increase in tensile strength to 255 MPa and a significant improvement in ductility, reaching an A5 value of 8.7%. The best mechanical properties were observed in Billet 3, which had the finest microstructure (Figure 3b and Figure 6b, i.e., 170 μm).

## 4. Discussion

The selection of an appropriate grain refiner should be based on a detailed analysis of the process requirements and the desired properties of the final product. Therefore, it is essential to determine the type and quantity of the grain refiner for a given alloy. This becomes particularly challenging in direct chill (DC) casting of billets made from alloys containing Zr, Cr, Li, or higher Si content. The challenge lies in optimising the injection points and the feed rate of the refining rods, which depend on various process parameters such as the metal temperature, the metal flow rate in the trough, as well as the design and arrangement of the refiners and filters. Our results confirm the significance of these parameters, particularly in the presence of 0.15% Zr, which strongly affects grain refinement efficiency.

After introducing TiB_2_-based refiner rods, sufficient time must be allowed for melting and even distribution of TiB_2_ particles. It is crucial that the refiner is activated before the molten metal reaches the mould (crystallizer). This study confirms previous findings by Read [10] and Bunn et al. [11] that longer holding times lead to grain coarsening, especially in Zr-containing alloys.

An analysis of the chemical composition of the AlSiMgCuZr alloy after adding the Al-5Ti-1B master alloy showed that the Ti and B content decreased over time, and even after a short time their concentration in the alloy should be maintained at a higher level. Such time-dependent reduction in active elements aligns with the poisoning mechanism proposed in [19], where TiB_2_ becomes chemically altered by Zr diffusion. The study revealed that the presence of 0.15% Zr promotes grain growth, leading to a coarse-grained and fully columnar grain structure in the AlSiMgCu alloy with an extended retention time of the commercial Al-5Ti-1B grain refiner in the molten metal, confirming the literature data [4,5,6,7,8,9,10]. Compared to similar studies, such as those by Zhang et al. [29], our STEM-EDS mapping demonstrates Zr enrichment at the TiB_2_ particle interface, validating the Zr-Contained Multi-Layers (ZCMLs) hypothesis as a potential cause for reduced nucleation potential.

The results showed that the addition of 60 ppm B in the form of AlTi5B1 before casting initially reduced the grain size at the beginning of the 1-metre billet to 350 µm (Figure 1 and Figure 4). However, at the end of the 2-metre billet, approximately 30 min after casting, a significant increase in grain size was observed, aligning with the findings reported by P.J. Read [10]. This observation supports the theory that prolonged interaction between Zr and the TiB_2_ refiner significantly weakens its nucleation capacity. The presence of TiB_2_ clusters in the Zr-enriched areas at the end of the billet 1 (Figure 8) confirms the poisoning effect, which may result from chemical and structural modifications of TiB_2_ particle surface in the Al-Ti-B master alloy, ultimately reducing their ability to heterogeneously nucleate α-Al grains [4,8,14,15,16,17,18,19]. This finding is consistent with simulation results from Pang et al. [20], who showed that Zr modifies the surface chemistry of Al_3_Ti, decreasing its effectiveness in grain initiation. As a result of the analyses carried out using (S)TEM, it can be concluded that Zr is present in TiB_2_ particles. From the linear analysis profile, it can be seen that Zr is deposited on the outer surface of the particle (Figure 10, Figure 11 and Figure 12). This distribution pattern was also identified by Wu et al. [30], who noted that Zr tends to accumulate at the TiB_2_ surface, altering its interfacial energy.

It has been demonstrated that increasing the addition of Al-5Ti-1B above 60 ppm B (300 ppm Ti) in an AlSiMgCu alloy containing Zr and introducing refining bars into the trough during casting of billets 2 and 3 improved the grain refinement efficiency (Figure 2, Figure 3, Figure 5 and Figure 6), which is in line with the published results [4,16,31].

This suggests that dynamic addition of grain refiner during casting may counteract some of the poisoning effects, likely by ensuring a continuous supply of active TiB_2_ particles to the melt.

Although billets are semi-finished products intended for extrusion, the conducted tests on billets with different grain structures clearly indicate a significant influence of grain size on the mechanical properties of the material (Figure 14). This is in agreement with the Hall–Petch effect, which predicts increased strength and ductility with grain size reduction.

The improvement of the structural uniformity in cross-section and along the billet length, in particular the reduction in the average grain size, significantly impacts subsequent hot forming processes. Extruding billets with a smaller average grain size requires less pressing force, ensures greater process stability and enables higher profile extrusion speeds. Our findings are consistent with observations reported by Grandfield and Eskin [17], who linked refined grain structure with increased extrudability and reduced defect rates.

## 5. Conclusions

This study investigated the effects of holding time and the amount of Al-Ti-B grain refiner on the structure and properties (hardness distribution across the diameter) of AlMgSi(Cu) billets containing Zr. The key conclusions are as follows:

- It has been demonstrated that Ti and B content decreases over time, and the final Ti and B content in the billets was approximately 20–50% lower than expected.
- The presence of 0.15 wt.% Zr, in combination with the Al-5Ti-1B grain refiner in the molten metal, causes grain coarsening over time, thereby disrupting the refiner’s effectiveness.
- Analysis of the microstructure revealed the presence of TiB_2_ metallic compounds derived from the grain refiner in the presence of Zr.
- Increasing the Al-5Ti-1B addition to 80 ppm B (400 ppm Ti) in an AlSiMgCu alloy containing 0.15 wt.% Zr significantly improves grain refinement.
- The Al-5Ti-1B grain refiner should be added in the form of a rod directly before casting to minimise the Zr poisoning effect, which enhances the nucleation efficiency of α(Al) grains and allows the desired grain size in billets to be achieved.
- These results confirm previous literature findings and support the theory of Zr poisoning via modification of TiB_2_ particles.

## Figures and Tables

**Table 1:** Casting parameters for 7-inch billets.

Parameter	Value		
Metal temperature in the transition plate [°C]	690–670	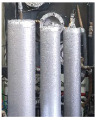	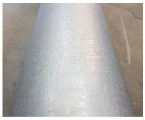
Casting speed [mm/min]	66–73		
Cooling water flow rate [L/min]	95–105		

## Data Availability

The original contributions presented in this study are included in the article. Further inquiries can be directed to the corresponding author.

## References

[1] Cibula A. (1950). The Mechanism of Grain Refinement of Sand Castings in Aluminum Alloys. J. Inst. Met..

[2] Cibula A. (1951). The Grain Refinement of Aluminium Alloy Castings by Additions of Titanium and Boron. J. Inst. Met..

[3] Greer L. (2016). Overview: Application of Heterogeneous Nucleation in Grain-Refining of Metals. J. Chem. Phys..

[4] Jones G.P., Pearson J. (1976). Factors Affecting the Grain-Refinement of Aluminum Using Titanium and Boron Additives. Metall. Trans. B.

[5] Birch M.E.J., Fisher P., Shepard T. (1986). Grain Refining of Commercial Aluminium Alloys with Titanium Boron Aluminium. Conference on Aluminium Technology 86.

[6] Birch M.E.J., Cowell A.J.J. (1988). Grain Refinement of Aluminium Alloys Containing Chromium and Zirconium. Conference on Solidification Processing.

[7] Abdel-Hamid A.A. (1989). Effect of Other Elements on the Grain Refinement of Al by Ti or Ti and B. Int. J. Mater. Res..

[8] Johnsson M. (1994). Influence of Zr on the Grain Refinement of Aluminium. Int. J. Mater. Res..

[9] Kearns M.A., Cooper P. (1997). Effects of Solutes on Grain Refinement of Selected Wrought Aluminium Alloys. Mater. Sci. Technol..

[10] Read P.J., Ferrolegeringar AG Zurych (1996). Stopy Przejściowe Aluminium w Odlewnictwie. Proceedings of the IMN OML Conference on Aluminium Technology 96.

[11] Bunn A.M., Schumacher P., Kearns M.A., Boothroyd C.B., Greer A.L. (1999). Grain Refinement by Al-Ti-B Alloys in Aluminium Melts: A Study of the Mechanisms of Poisoning by Zirconium. Mater. Sci. Technol..

[12] Spittle J.A., Sadli S. (1995). The Influence of Zirconium and Chromium on the Grain Refining Efficiency of Al-Ti-B Inoculants. Cast Metals.

[13] Qiu D., Taylor J.A., Zhang M.-X. (2010). Understanding the Co-Poisoning Effect of Zr and Ti on the Grain Refinement of Cast Aluminum Alloys. Metall. Mater. Trans. A.

[14] McCartney D.G. (1989). Grain Refining of Aluminium and Its Alloys Using Inoculants. Int. Mater. Rev..

[15] Rao A.A., Murty B.S., Chakraborty M. (1996). Influence of Chromium and Impurities on the Grain-Refining Behavior of Aluminum. Metall. Mater. Trans. A.

[16] Murty B.S., Kori S.A., Chakraborty M. (2002). Grain Refinement of Aluminium and Its Alloys by Heterogeneous Nucleation and Alloying. Int. Mater. Rev..

[17] Grandfield J.F., Eskin D.G., Bainbridge I.F. (2013). Direct-Chill Casting of Light Alloys.

[18] Schumacher P., Cizek P., Bunn A., Grandfield J.F., Eskin D.G. (2016). Zr-Poisoning of Grain Refiner Particles Studied in Al-Ni-Zr Amorphous Alloys. Essential Readings in Light Metals.

[19] Wang Y., Fang C.M., Zhou L., Hashimoto T., Zhou X., Ramasse Q.M., Fan Z. (2019). Mechanism for Zr Poisoning of Al-Ti-B Based Grain Refiners. Acta Mater..

[20] Pang X., Yang L., Yang J., Pang M., Xu Z., Li A., Wei B., Tang H. (2023). Understanding the poisoning mechanisms of Si and Zr atoms on L1_2_ Al_3_Ti (111) surface: A first-principles investigation. Vacuum.

[21] Whitehead A.J., Danilak S.A., Granger D.A., Huglen R. (1997). The Development of a Commercial Al-3%Ti-0.15%C Grain Refining Master Alloy. Proceedings of Light Metals 1997.

[22] Schneider W., Kearns M.A., McGarry M.J., Whitehead A.J., Grandfield J.F., Eskin D.G. (2016). A Comparison of the Behaviour of AlTiB and AlTiC Grain Refiners. Essential Readings in Light Metals.

[23] Birol Y. (2006). Grain Refining Efficiency of Al–Ti–C Alloys. J. Alloy. Compd..

[24] Kumar G.S.V., Murty B.S., Chakraborty M. (2005). Development of Al-Ti-C Grain Refiners and Study of Their Grain Refining Efficiency on Al and Al-7Si Alloy. J. Alloy. Compd..

[25] Ding H.M., Liu X.F., Yu L.N. (2007). Influence of Zirconium on Grain Refining Efficiency of Al-Ti-C Master Alloys. J. Mater. Sci..

[26] Li D., Wang Y., Zhang Y., Liu Y., Liu J., Chen Z. (2023). An Anti Si/Zr-Poisoning Strategy of Al Grain Refinement by the Evolving Effect of Doped Complex. Acta Mater..

[27] Fang X., Li J., Zhang W., Wang H., Liu Y., Chen Q. (2024). Simultaneous refinement of α-Al and modification of Si in Al–Si alloy achieved via the addition of Y and Zr. J. Mater. Res. Technol..

[28] (2020). Metallic Materials—Tensile Testing—Part 1: Method of Test at Room Temperature.

[29] Zhang L., Yang L., Zhao J., Shen Z., Li Q., Jiang H. (2024). A Novel Insight Toward Zr Poisoning on Grain Refinement of Al–5Ti–1B and Its Solution. Metall. Mater. Trans. B.

[30] Wu J., Ruan Q., Chen S., Meng C., Xu Z., Wei C., Tang H., Wang J. (2022). Insights into Poisoning Mechanism of Zr by First Principle Calculation on AdhesionWork and Adsorption Energy between TiB2, Al3Ti, and Al3Zr. Metals.

[31] Rao A.A., Murty B.S., Chakraborty M. (1997). Role of Zirconium and Impurities in Grain Refinement of Aluminium with Al-Ti-B. Mater. Sci. Technol..

